# Behcet’s disease and multiple arterial aneurysms: 4 case reports

**DOI:** 10.1093/jscr/rjab200

**Published:** 2021-05-27

**Authors:** Javad Salimi, Zahra Omrani, Roozbeh Cheraghali

**Affiliations:** Vascular and Endovascular Surgery, Liver Transplantation Program, Tehran University of Medical Sciences, Tehran, Iran; Department of Surgery, Iran University of Medical Sciences (IUMS), Tehran, Iran; Vascular and Endovascular Surgery, Tehran University of Medical Sciences (TUMS), Tehran, Iran

## Abstract

Behcet’s disease (BD) is a vasculitis of unknown origin. BD is rare in Western countries and the southern hemisphere and is mainly seen in countries along the Silk Road such as the Middle East and Mediterranean regions including Iran. We report four interesting cases of BD with multiple aneurysms three of which had subclavian aneurysms. We chose different surgical approaches for each of these patients due to the different presentations and unique circumstances of each case. Endovascular stent graft used for patients and we discussed open vs. endovascular treatment for BD aneurysms in the discussion.

## INTRODUCTION

The incidence of vascular involvement in patients with Behcet’s disease (BD) ranges from 7 to 38% [1]. Vascular involvement can include both arteries and veins, with lesions ranging from arterial occlusions and aneurysms to superficial thrombophlebitis [2, 3]. We report four cases of BD with vascular involvement, all of whom had more than one aneurysm. Three patients underwent endovascular stent graft except one who refused surgical treatment and was followed up for a year.

**Figure 1:**
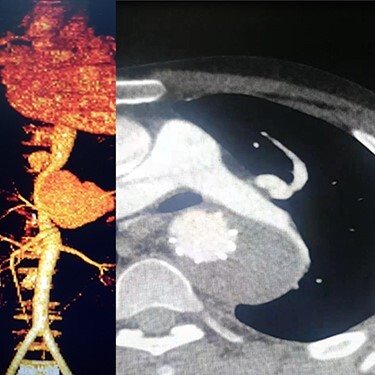
(**A**) CTA of the left subclavian aneurysm (right), (**B**) CTA of thoraco abdomen aneurysm (left).

Case 1 — A 30-year-old man who was a known case of BD and was diagnosed 5 years prior to admission to our center. The patient was admitted to our hospital with progressive chest and left upper extremity pain for 4 months. Chest computed tomography (CT) scan revealed a left saccular subclavian aneurysm (Fig. 1A) with no sign of rupture or pleural effusion. After 48 hours of admission, he suffered from severe abdominal pain, hypotension and tachycardia. After resuscitation, thoracoabdominal CT angiogram (CTA) was conducted which showed an aneurysm of the thoracoabdominal aorta (Fig. 1B) and was ruptured near Celiac artery origin. Endoxan (1 gr) and Methyl prednisolon pulse (1 gr daily up to 3 days) were injected. Emergency thoracic endo vascular aortic repair (TEVAR) was performed and he became stable after the procedure. Tablet of Prednisolon 5 mg was prescribed twice daily. In follow-up angiography, the subclavian artery was ligated after the origin of the aortic arch and the aneurysm was thrombosed; however, since his arm was viable, conservative management was selected. After 1 year, he had no disabling abnormality on physical examination of his arm and no sign of endoleak was identified in the follow-up CTA.

**Figure 2:**
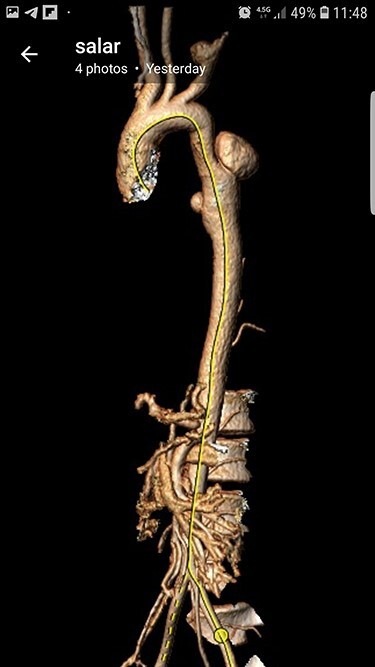
Preoperative CTA.

**Figure 3:**
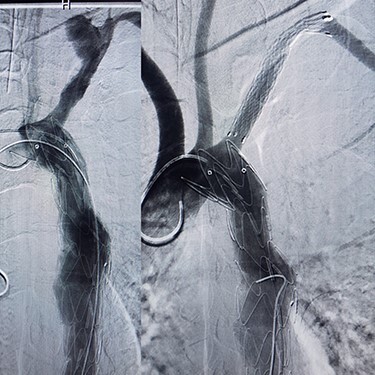
(**A**) Angiography after stent-graft placement of left subclavian (right), (**B**) thoracic aorta (left).

Case 2 — A 32-year-old man with known BD for years, presented to the emergency department with chest pain. We identified left subclavian and thoracic aorta saccular aneurysms on CTA (Fig. 2). Endoxan (1 gr) and Methyl prednisolon pulse (1 gr daily up to 3 days) were injected. The patient underwent a subclavian stent-graft (Fig. 3A) and elective TEVAR (Fig. 3B). Immediate follow-up angiogram revealed the luminal aperture was obtained and it was observed that the pseudoaneurysm remained occluded without a sign of leakage or endoleak. Tablet of prednisolon (5 mg) was prescribed twice daily.

**Figure 4:**
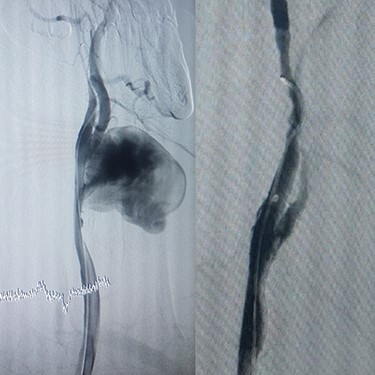
(**A**) Carotid angiography before stent-graft placement (left), (**B**) after stent-graft placement (right).

Case 3 – A 44-year-old man, known case of BD, who underwent open surgery for right common iliac artery aneurysm 1 year ago. The patient was referred to our hospital with a huge pulsatile neck mass. The right femoral artery pulse was absent in physical examination. CTA showed the iliac graft was thrombosed, but his lower limb did not have symptoms of ischemia. Corticosteroid therapy was prescribed before starting the procedure. In addition, a left carotid artery aneurysm was identified (Fig. 4A), so a stent-graft was inserted in the left common carotid (Fig. 4B). The pulsation and bruit of the mass disappeared immediately after the procedure and patient was discharged without complications. After 1-year follow-up, there was no sign of pseudo-aneurysm and the stent-graft was open.

**Figure 5:**
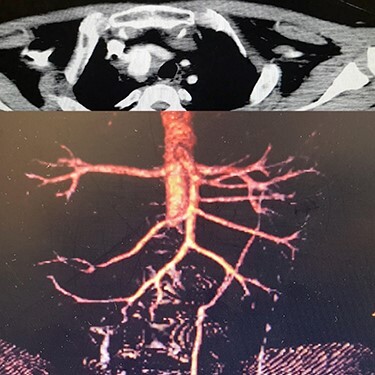
(**A**) Infra renal aortic thrombosis (down), (**B**) right subclavian aneurysm (up).

Case 4 – A 32-year-old man who was a known case of BD presented to the emergency department with a complaint of abdominal pain. In the abdominal CT scan, he had a thrombosed abdominal infrarenal aortic aneurysm (Fig. 5A). Heparin therapy was started. A huge right subclavian artery aneurysm was found in CTA (Fig. 5B). We talked to the patient about endovascular repair of the subclavian aneurysm but he refused the surgical treatment. Tablet of prednisolon (5 mg) twice a day was prescribed. In 1-year follow-up, the aneurysm size of the subclavian artery did not expand and his lower limbs were not symptomatic.

## DISCUSSION

Vascular involvement is a well-known complication of BD which is seen in up to 40% of patients and is considered to be a poor prognostic factor [4, 5]. Arterial involvement is less frequent in comparison with venous involvement and arterial aneurysm is more commonly reported than arterial occlusion [6].

The aorta is the most common site of aneurysm formation, with pulmonary, femoral, popliteal, brachial and iliac arteries also have been reported in descending order of frequency [7–10]. Interestingly, although subclavian artery is not a common site for aneurysm formation; herein, we report three subclavian aneurysms in four cases of BD. In one case, the aneurysm was thrombosed with no clinical symptoms, the patient was followed by antiagregant therapy. A stent graft was placed in the second patient, the third patient was treated medically and no intervention was performed because he refused to accept surgery. In this series of BD patients with arterial aneurysms, all the aneurysms were saccular and pseudoaneurysm. Our findings are in agreement with Matsumoto *et al.* [11] who also stated most BD aneurysms are pseudo-aneurysms. The anastomotic pseudo-aneurysm formation is a life threatening postoperative complication that may occur in some BD patients, although its incidence has never been elucidated.

In our hospital, endovascular techniques is our preferred choice of procedure, as the repair process in Behcet’s patients is challenging. The anastomotic fragility in patients with BD might be attributable to the weakening of the arterial wall caused by fulminant inflammation. It is difficult to determine the suitable site for anastomosis of a graft with arterial wall, preoperatively or intra-operatively. Macroscopic examination alone might not be sufficient to identify a disease-free portion of the artery. Suture lines of the anastomotic site in open surgery would form pseudo-aneurysm again in both autonomous or synthetic grafts. Also, due to all the points mentioned above, the repair of the sheath insertion site should be performed with caution.

Koksoy *et al*. [10] encountered post-operative pseudoaneurysms in 7/32 (24%) of their patients. Nowadays, stent-grafts are an alternative treatment for aortic and arterial aneurysms in patients with BD and they can be effective, safe, and associated with an acceptable vascular complication rate and low mortality. [12].

## CONCLUSION

As many BD have multiple vascular (venous or arterial) involvements, a thorough and comprehensive examination and diagnostic work up should be considered in the patients with BD. Endovascular therapy (stent-graft) in patients with BD appears to be a promising and effective alternative management option in comparison with open surgical procedures.

## References

[1] Sakane T, Takeno M, Suzuki N, Inaba G. Behcet's disease. N Engl J Med 1999;341:1284–91. 10528040 10.1056/NEJM199910213411707

[2] Zhang S-H, Zhang F-X. Behcet’s disease with recurrent thoracic aortic aneurysm combined with femoral artery aneurysm: a case report and literature review. J Cardiothorac Surg 2017;12:79. 28874203 10.1186/s13019-017-0644-y PMC5585960

[3] Memetoglu ME, Kalkan A. Behçet’s disease with aneurysm of internal iliac artery and percutaneous treatment. Interact Cardiovasc Thorac Surg 2012;14:372–4. 22159243 10.1093/icvts/ivr041 PMC3290367

[4] Ulusan S, Karadag M, Tasar M, Kalender O. Darcin Behcet’s disease and cardiovascular involvement: our experience of asymptomatic Behcet’s patients. Cardiovasc J Afr 2014;25:63–6. 24844550 10.5830/CVJA-2014-003 PMC4026764

[5] Fei Y, Li X, Lin S, Song X, Wu Q, Zhu Y, et al. Major vascular involvement in Behcet’s disease: a retrospective study of 796 patients. Clin Rheumatol 2013;32:845–52. 23443336 10.1007/s10067-013-2205-7

[6] El-Ghobashy N . Kamal El-Garf Marwa Abdo, arterial aneurysms in Behçet’s disease patients: frequency, clinical characteristics and long-term outcome. Egypt Rheumatol 2018;41:309–12.

[7] Sidawy AN, Perler BA, et al. Rutherford’s Vascular Surgery and Endovascular Therapy Book, Elsevier, 9th edn. 2309.

[8] Morata AR, Conde AH, Cosme CC, Ramirez SG, Medialdea RG. Atypical vascular involvement in a case of Behcet’s disease. Case Rep Surg 2012;2012:848101. 10.1155/2012/848101 PMC3514815 23227411

[9] Christensen PA, Tvedegaard E, Strandgaard S, Thomsen BS. Behcet’ s syndrome presenting with peripheral arterial aneurysms. Scand J Rheumatol 1997;26:386–8. 9385353 10.3109/03009749709065705

[10] Koksoy C, Alacayir I, Bengisun U, Uncu H, Anadol E, Gyedu A. Surgical treatment of peripheral aneurysms in patients with Behcet’s disease. Eur J Vasc Endovasc Surg 2011;42:525–30. 21641238 10.1016/j.ejvs.2011.05.010

[11] Matsumoto T, Uekusa T, Fukuda Y. Vasculo-Behcet’ sdisease: funding a pathologic study of eight cases. Hum Pathol 1991;22:45–51. 1985077 10.1016/0046-8177(91)90060-3

[12] Kim WH, Choi D, Kim JS, Ko YG, Jang Y, Shim WH. Effectiveness and safety of endovascular aneurysm treatment in patients with vasculo-Behcet disease. J Endo Vasc Ther 2009;16:631–6. 10.1583/09-2812.1 19842735

